# Clinical Performance of Flash Glucose Monitoring System in Patients with Liver Cirrhosis and Diabetes Mellitus

**DOI:** 10.1038/s41598-020-64141-x

**Published:** 2020-05-04

**Authors:** Dalila Costa, Joana Lourenço, Ana Margarida Monteiro, Beatriz Castro, Patricia Oliveira, Maria Carmo Tinoco, Vera Fernandes, Olinda Marques, Raquel Gonçalves, Carla Rolanda

**Affiliations:** 1 Life and Health Sciences Research Institute (ICVS), School of Medicine, University of Minho, Braga, Portugal; 2 Gastroenterology Department, Braga Hospital, Braga, Portugal; 3 Endocrinology Department, Braga Hospital, Braga, Portugal

**Keywords:** Liver cirrhosis, Type 2 diabetes

## Abstract

Flash glucose monitoring system (FGMS) is an improved subset of continuous glucose monitoring with a recognized effectiveness on glycemic control, though validation in patients with Liver Cirrhosis (LC) is lacking. To evaluate the accuracy of FGMS in patients with Type 2 Diabetes Mellitus (DM) and LC, a prospective, case-control study was performed in 61 ambulatory patients with LC and DM (LC group, n = 31) or DM (Control group, n = 30). During 14 days, patients performed 4 assessments per day of self-monitoring of blood glucose (SMBG, reference value) followed by FGMS scanning. There were 2567 paired SMBG and FGMS values used in the accuracy analysis, with an overall mean absolute relative difference (MARD) of 12.68% in the LC group and 10.55% in the control group (p < 0,001). In patients with LC, the percentage of readings within Consensus Consensus Error Grid analysis Zone A and A + B were 80.36% and 99,26%, respectively. Sensor clinical accuracy was not affected by factors such as body mass index, age, gender, Child-Pugh score or edematoascitic decompensation. This is the first study to approach FGMS clinical accuracy in LC, revealing a potential usability of this system to monitor glycemic control in this population.

## Introduction

Type 2 Diabetes Mellitus (DM) has been considered a Public Health challenge due to its significant impact on mortality and morbidity worldwide^1^. The interplay between DM or even impaired glucose tolerance and Chronic Liver Disease (CLD) is now well established in the literature^2–7^. Even though DM is a well-recognized metabolic risk factor for CLD^8,9^, the liver also plays a primary role in carbohydrate metabolism, thus DM can also occur as a result of CLD progression^2,9^. In fact, among patients with liver cirrhosis (LC), the prevalence of DM ranges from 7 to 60%, according to different authors^2,3,7^. Moreover, DM increases the risk of cirrhosis complications and mortality by liver failure^4,6^.

Diagnosis and management of DM in patients with CLD can be extremely challenging^10,11^. Despite the limitations described in the literature, the current options for their glycemic control monitoring are the same as the recommended for DM population in general^11,12^.

Glycated Hemoglobin (HbA1c) is routinely used as a standard measure for long-term glucose control^13^ and estimates the average glycemic status over the last 3 months^7^. This measurement frequently underestimates glycemic control in LC, probably as a result of shortened red blood cells half-life due to hypersplenism, blood loss into the gastrointestinal tract or iron deficiency^7,12–15^. Fructosamine also provides an estimation of average blood glucose during the past 2 weeks^15^. Since it represents stable ketoamines formed from glycated plasmatic proteins, this may be potentially altered by hypoalbuminemia and hyperbilirubinemia, commonly observed in CLD^7,16^. Glycated albumin is fructosamine main fraction, therefore it has similar limitations^7^. CLD-HbA1c formula suggested by Koga *et al*.^17^ combines HbA1c with glycated albumin. Although it appears to be a promising tool due to its high correlation with estimated HbA1C levels, there is insufficient evidence to support its use in clinical practice^7,17^.

Self-monitoring of blood glucose (SMBG) has been suggested in CLD^6,7^. It provides an accurate estimation of the real blood glucose control and is not affected by the above mentioned limitations^18,19^. However, there are a few physiologic and pharmacologic factors that may interfere with glucose readings^20^. Moreover, SMBG relies on meter accuracy, patients’ adherence and may not reflect glycemic fluctuations^7,12^.

Continuous glucose monitoring (CGM) is a method based on interstitial glucose measurement through a cutaneous sensor^6^. Flash glucose monitoring system (FGMS) is a subset of CGM, with the advantage of not requiring user calibration and displaying the information using graphs and trend arrows^21^. It is indicated for individuals with DM over 4 years old, including pregnant women, and has a proven impact on adherence, glycemic control and DM prognosis^22–24^.

The blood glucose dynamics in CLD has been scarcely studied. Even so, and especially in LC, marked glycemia fluctuations appear to be frequent, with a higher risk of severe hypoglycemic events, postprandial hyperglycemia and nocturnal hypoglycemia^3,7,19^, which are easily undervalued by standard monitoring tools. Therefore, some authors defend the potential applicability of CGM in CLD, since it is not conditioned by the limitations described for standard glucose monitoring methods^6,7,19^. However, there is insufficient literature about the utility of CGM in these population, particularly the new subset FGMS, which has not been validated as a suitable option for monitoring DM in patients with LC.

## Materials and Methods

### Patients and data collection

A prospective, case-control and single-center study was performed in ambulatory patients with clinical and/or histological diagnosis of LC and analytical diagnosis of DM, evaluated between January and October 2018 at the Gastroenterology Department of Braga Hospital.

Patients meeting any of the following criteria were excluded: active alcohol abuse, oncological disease (particularly hepatocellular carcinoma), portal vein thrombosis, chronic pancreatitis, congestive heart failure, chronic kidney disease, Human Immunodeficiency Virus infection, Mycobacterium tuberculosis and Hepatitis C virus (HCV) infection under pharmacological treatment, corticosteroids therapy, extended hospitalization during the study period and psychic/cognitive state alteration.

A sample size of 79 was sought, considering the criteria of 95% Confidence Level and 5% Confidence Interval applied by the Creative Research Systems software, available online. Since this sample could not be achieved, in order to minimize the loss of representativeness, a control group was created with patients with DM evaluated at the Endocrinology Department of Braga Hospital, without evidence of CLD (excluded based on imaging and biochemical evaluation). Patients were paired in a ratio 1:1, using the tool “propensity score matching” from Statistical Package for the Social Sciences (SPSS Inc., Chicago, Illinois, USA), according to predefined baseline characteristics (gender, age, BMI, insulin use and HbA1c), selected to exclude any confounding factors. The exclusion criteria previously described for the LC group were also applied.

Medical records were reviewed. The following parameters were considered for analysis: sociodemographic (age, gender), clinical (Cirrhosis etiology, Child-Pugh, Model for End-Stage Liver Disease the serum sodium incorporated (MELD-Na) score, edematoascitic decompensation, Body Mass Index (BMI), comorbidities and medication) and recently collected biochemical data (hemogram, liver and kidney biochemistry, ionogram).

Written informed consent was obtained from all patients. The study was conducted in accordance with the Declaration of Helsinki and was approved by the Ethics Committee of Braga Hospital (Approval number: 116/2018).

### Glycemic control monitoring tools

Subcutaneous interstitial glucose levels were monitored using the FGMS FreeStyle Libre system (Abbott Diabetes Care, Alameda, CA). This tool includes a reader device and a small disposable sensor, applied on the back of the upper arm for up to 14 days, according to the manufacturer’s direction. It is factory-calibrated and has no automatic alarms. Data are transferred to the reader when it is brought into close proximity to the sensor, displaying current sensor glucose level. For a complete 24-hour glycemic profile, a scan once every 8 hours was required. Data are automatically stored on the sensor and can be uploaded from the reader, using the device software to generate summary glucose reports. Four sensor lots were used throughout the study, which is sufficient to demonstrate the performance of reagent systems across multiple production lots^25^. As recommended by Messer *et al*.^26^, in order to enhance sensor skin adhesion throughout device lifespan, kinesiology tape was applied over each participant’s sensor. It protects against water, increased perspiration and movement.

SMBG was achieved by finger-prick testing of capillary blood using the same reader of FGMS (Abbott Diabetes Care, Alameda, CA), in order to reduce the variability between devices. Glucose test strips (FreeStyle Precision) and lancets (Abbott Single Fire) were provided to the participants.

### Study design

There were two scheduled in-clinic visits. At the first visit (day 1), baseline HbA1c (HbA1cD1) was collected and 1 FGMS sensor per participant was inserted. Throughout the next 14 days, participants were asked to perform at least 4 assessments per day of SMBG, each followed 5 minutes later^27^ by FGMS sensor scanning. For each patient, measurements were scheduled upon waking, 2 hours after the beginning of lunch, 2 hours after the beginning of dinner and at bedtime. Though results were automatically stored and uploaded from the reader at the end of the study, individuals were also asked to register the data on a given table. Patients were advised to maintain their established diabetes management plan.

A second visit (day 15) was programmed to deliver all the materials and upload the results. Between in-clinic visits, patients were contacted to clarify any doubt. Sensors that were accidentally dislodged within the first week of use were replaced. Sensors that were dislodged after that time were not replaced.

### Statistical analysis

Statistical analysis was performed using SPSS software (Chicago, Illinois, USA), and MATLAB software (Mathworks Inc.).

Descriptive data were summarized using the appropriate statistical tools, given the nature of the variables involved. Normal distribution was assessed by Kolmogorov-Smirnov test. Student t or Mann-Whitney tests were performed to compare the distribution of independent continuous variables. Spearman correlation was used to evaluate correlation between continuous variables. Chi-Square test was assessed to test the association between categorical variables. The level of significance was set at p < 0.05.

To evaluate the analytical accuracy of FGMS, Mean Absolute Relative Difference (MARD) was calculated using SMBG as a reference, according to the formula: (|SMBG − FMGS|)/SMBG × 100^25^. Ancona *et al*.^28^ suggested that a value below 14% represents acceptable accuracy, a value between 14% and 18% represents intermediate accuracy and a value above 18% represents poor accuracy. Linear Regression was performed to identify any predictors of MARD.

The Consensus and Clarke Error Grid Analysis (EGA) were used to assess the magnitude of clinical risk from inaccurate flash glucose readings (clinical accuracy). EGA, widely accepted tool for defining glucose meter’s accuracy, subdivides plotted results into 5 zones: A, less than 20% difference from reference values; B, difference greater than 20% but the resulting clinical decisions are uncritical; C, could cause an overcorrection of glycemia; D, represents a dangerous failure to detect and treat; E, leads to erroneous treatment^29^.

## Results

### Patients baseline characteristics

**Figure 1:**
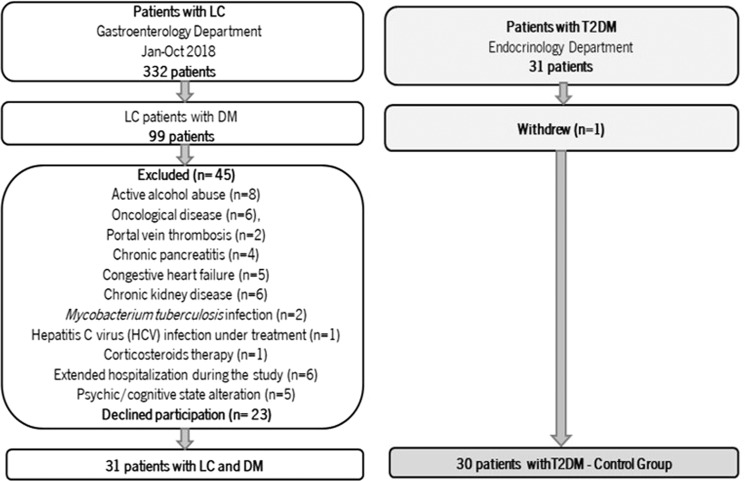
Study flow diagram. LC: Liver cirrhosis; T2DM: Type 2 Diabetes Mellitus; DM: Diabetes Mellitus; HCV: Hepatitis C Virus; Jan-Oct: January to October.

Over the study period, 62 participants were enrolled in the study, but ultimately only 61 patients were included in the analysis: 31 in LC group and 30 in the control group (Fig. 1). Considering the predefined baseline characteristics, no significant differences were revealed between the groups (Table 1). Additionally, all patients were on oral antidiabetic drug (OAD), mostly metformin (LC group: 76,7% v Control group: 77,4%; p = 0,994).

**Table 1:** Patients baseline characteristics.

	LC group	Control group	p value
**Gender, frequency (%)**			
Male	21 (67.7%)	15 (50.0%)	0.159
Female	10 (32.3%)	15 (50.0%)	
Age, years*^1^			
Mean ± SD	63 ± 10	64 ± 7	0.716
**BMI, kg/m**^2^*^2^			
Median (P25-P75)	30.49 (24.15–34.14)	27.22 (25.07–28.34)	0.360
**Insulin use, frequency (%)**			
yes	17 (54.8%)	16 (46.7%)	0.906
no	14 (45.2%)	14 (46.7%)	
HbA1cD1, %*^1^			
Mean ± SD	6.6 ± 1.4	7.1 ± 1.3	0.152

LC: Liver Cirrhosis; T2DM: Type 2 Diabetes Mellitus; BMI: Body Mass Index; HbA1cD1: glycated hemoglobin performed in Day 1 of study; Frequency: absolute frequency; %: relative frequency; SD: Standard Deviation; P25: Percentile 25; P75: Percentile 75; *^1^Independent samples Student t test. *^2^Mann-Whitney test. All other were analysed with Chi-Square test.

Regarding cirrhosis etiology, 64.5% (n = 20) were related to chronic alcohol abuse (CAA), followed by non-alcoholic steatohepatitis (NASH) in 19.4% (n = 6) and other causes (HCV, Hepatitis B Virus and primary biliary cirrhosis) in 16.1% (n = 5). Patients were graded Child-Pugh A (67.7%, n = 21) or B (32.3%, n = 10). The median prognostic MELD-Na score was 10. Edematoascitic decompensation was evident in 32.3% (n = 10) and 58,1% (n = 18) were on diuretics (furosemide and/or spironolactone).

### FGMS analytical accuracy

There were 2565 paired capillary blood glucose to sensor glucose values used in the accuracy analyses. There were no unexpected adverse device effects reported. 4 participants (6.56%) experienced anticipated side effects (1 minor bleeding at sensor application and 3 mild erythema and itching at sensor insertion site). Eight sensors were accidentally dislodged prior to the full lifetime (4 sensors were replaced). No significant differences were observed between the groups regarding side effects and dislodged sensors. Eleven pairs were excluded because the reference glucose result was beyond the System’s dynamic range (40–500 mg/dL).

The FGMS sensor results were highly correlated to capillary SMBG in LC patients (ρ = 0.930, p < 0.001) and in the control group (ρ = 0.929, p < 0.001). An overall MARD of 12.68% was obtained in the LC group and 10.55% in the control group, with a significant difference between the groups (p < 0.001). Sensor analytical accuracy remained acceptable across the 14 days and through different glucose levels (Table 2).

**Table 2:** FGMS analytical accuracy as a function of various factors.

	LC group		Control group		p value
	MARD, %	95% CI	MARD, %	95% CI	
Overall	12.68	12.14–13.22	10.55	10.06–11.02	<0.001*
First week	11.47	10.76–12.19	9.62	9.04–10.20	<0.001*
Second week	13.92	13.15–14.70	11.39	10.69–12.16	<0.001*
Below 80 mg/dL	11.52	6.35–14.68	8.62	6.45–10.74	0.950
80–120 mg/dL	12.51	11.78–13.24	9.37	8.61–10.20	<0.001*
Above 120 mg/dL	12.83	12.08–13.59	11.27	10.65–11.91	<0.001*

LC: Liver Cirrhosis; MARD, %: mean absolute relative difference in percentage; 95% CI: Confidence Interval at 95% confidence level. *Statistically significant variables. All variables were analysed with Mann-Whitney test.

In the LC group, male gender (p = 0.003), Child-Pugh B (p < 0.001), and edematoascitic decompensation (p < 0.001) were significantly associated with a higher MARD. Nevertheless, following linear regression analysis, only the presence of edematoascitic decompensation significantly influenced MARD value (B = 5,19; 95% CI 4,01–6,37; p < 0,001) (Table 3).

**Table 3:** Clinical factors affecting FGMS analytical accuracy in LC.

LC group (n = 31)	Univariable		Multivariable		p value
	MARD,%	p value	B	95% CI	
**Gender**					
Female	11.36	0.003			
Male	13.18				
**Age**					
< 60 years	12.71	0.629			
≥60 years	12.65				
**Obesity**					
No	12.25	0.051			
Yes	13.09				
**Edematoascitic decompensation**					
No	11.36	<0.001*	5.19	4,01–6.37	<0.001*
Yes	16.52				
**Child-Pugh**					
A	12.09	<0.001*			
B	14.38				

LC: Liver Cirrhosis; MARD, %: mean absolute relative difference in percentage; 95% CI: Confidence Interval at 95% confidence level. *Statistically significant variables. Univariable analysis was performed with Mann-Whitney test. Multivariable analysis was performed with Linear Regression.

### FGMS clinical accuracy

**Figure 2:**
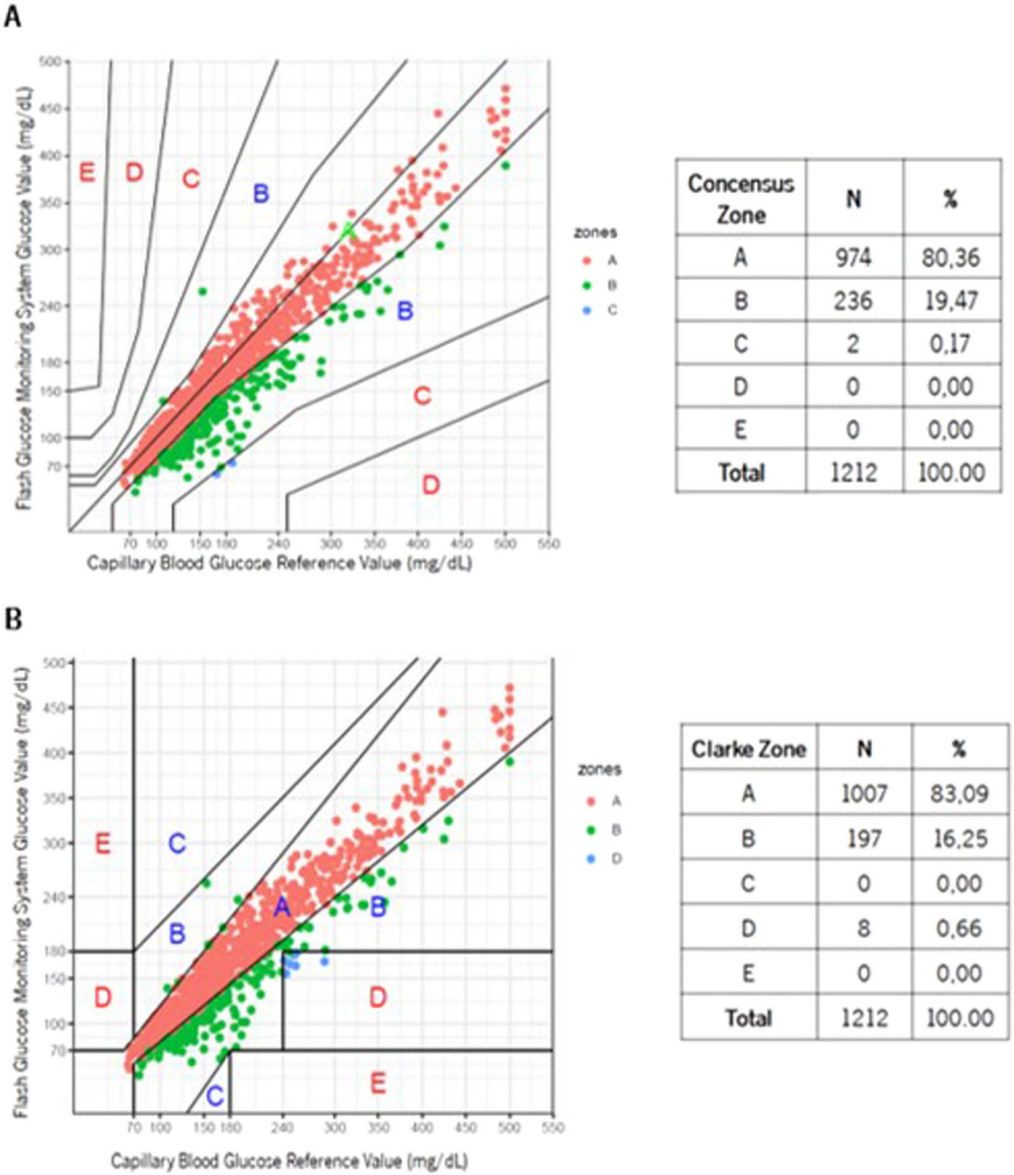
Liver Cirrhosis group Consensus (**A**) and Clarke (**B**) Error Grid Analysis comparing FGMS and SMBG.

**Figure 3:**
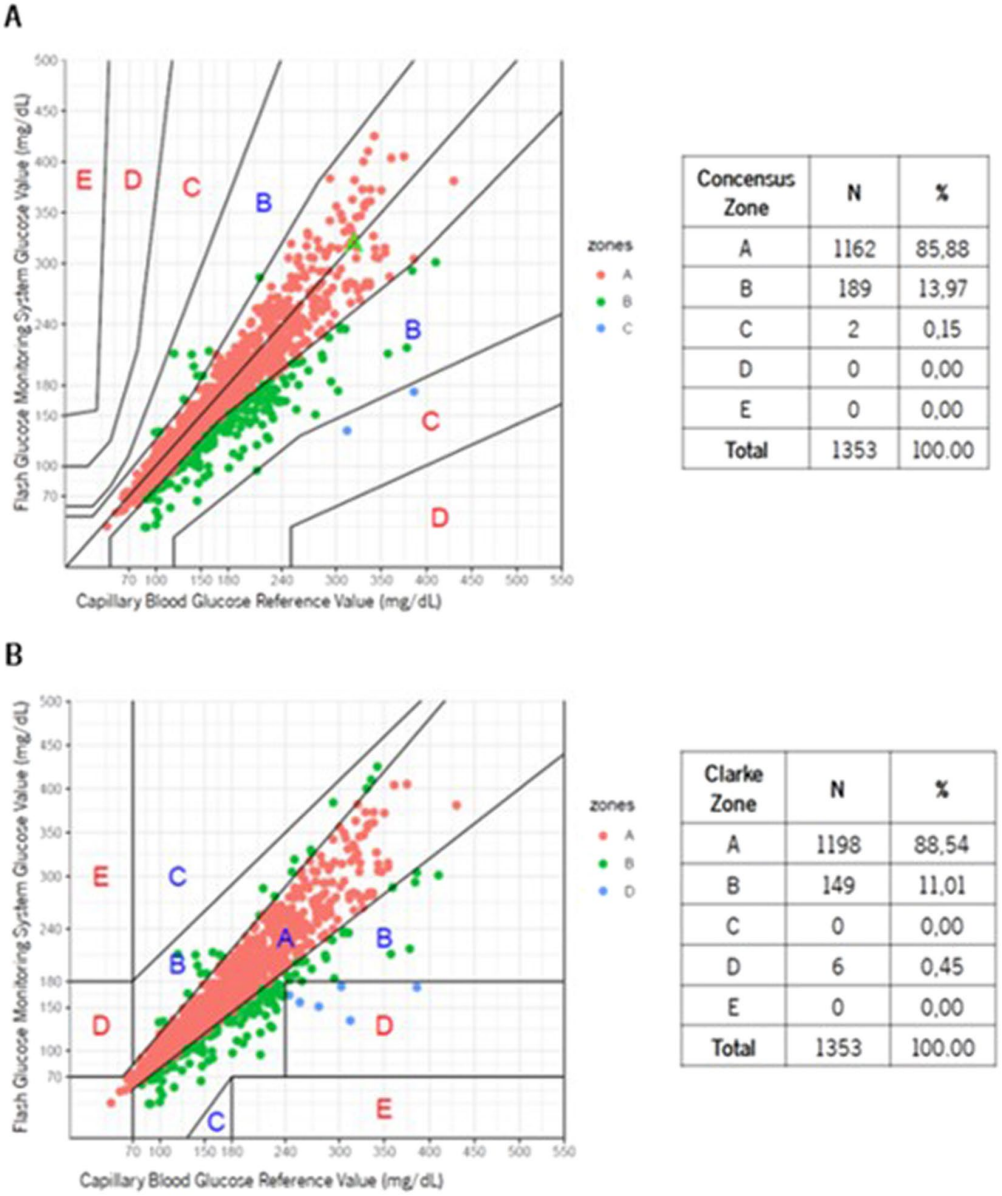
Control group Consensus (**A**) and Clarke (**B**) Error Grid Analysis comparing FGMS and SMBG.

In the LC group, Consensus EGA demonstrated 80.36% of results in Zone A and 99.83% in Zones A + B (Fig. 2A), whereas Clarke EGA demonstrated 83.09% of results in Zone A and 99.34% in Zones A + B (Fig. 2B). In the control group, Consensus EGA demonstrated 85.88% of results in Zone A and 99.85% in Zones A + B (Fig. 3A), whereas Clarke EGA demonstrated 88.54% of results in Zone A and 99.55% in Zones A + B (Fig. 3B).

Considering only the patients with LC, sensor clinical accuracy was not affected by factors such as gender, age, body mass index, Child-Pugh or edematoascitic decompensation, as the percentages of readings within Consensus Error Grid Zone A + B were persistently high (Table 4).

**Table 4:** Consensus EGA as a function of various factors in LC.

LC group (n = 31)	Consensus EGA Zone	
	Zone A, %	Zones A + B, %
**Gender**		
Female	84.52	100.00
Male	78.77	99.77
**Age**		
< 60 years	78.03	100.00
≥60 years	81.72	99.74
**Obesity**		
No	84.28	99.67
Yes	76.54	100.00
**Edematoascitic decompensation**		
No	85.94	99.78
Yes	64.10	100.00
**Child-Pugh**		
A	82.77	99.78
B	73.10	100.00

LC: Liver Cirrhosis; Consensus EGA: Consensus Error Grid Analysis; %: percentage of results within zone.

## Discussion

Monitoring DM in patients with LC can be extremely challenging^10,11^. Since standard monitoring tools, such as HbA1c and capillary blood glucose, reveal significant limitations in LC, the need for other methods emerges. To our knowledge, this is the first study to evaluate the performance of FGMS to monitor DM in patients with LC.

Study results showed a strong agreement between FGMS readings and SMBG in LC. The use of capillary SMBG data as a reference allowed the evaluation of real-life accuracy of FGMS under normal daily use. An overall MARD of 12.68% was achieved in the LC group and 10.55% in the control group, which are good performances and similar to other studies with different target populations (10.0–13.9%)^25,28–31^.

Sensor analytical accuracy remained acceptable (below 14%) during the period evaluation and through different blood glucose intervals in LC, as also revealed by Bailey *et al*.^25^. Nevertheless, a higher MARD was perceived in the second week and a wide range of MARD was observed in hypoglycemia. These results are not consistent in the literature. Some authors revealed a significant MARD decrease in second week of use and less accuracy during hypoglycemia^25,30,31^. It should be emphasized that studies are not completely comparable, either by study design or because the same percentage change in MARD in two studies may not correspond to the same glycemic variation. This also justifies MARDs wide percentage range. Therefore, the best assessment should be against our control group, which revealed the same results.

Although an acceptable analytical accuracy was achieved in patients with LC, MARD was persistently higher than the control group. We presumed that fluid overload could possibly conditioned glucose concentration in the interstitial fluid, since cirrhosis is characterized by a permanent hyperdynamic circulation and a fluid retention state. This study confirmed that the presence of edematoascitic decompensation has a significant impact on FGMS’s analytical accuracy, resulting in a higher MARD value and an intermediate accuracy.

Sensor clinical accuracy was also attained in LC, 80.36% of results were placed in Zone A and 99.83% in Zones A + B of Consensus EGA. Data was supported by Clarke EGA and similar results were achieved in the control group. Bailey and colleagues found a very similar accuracy in the first study published approaching the usability of FGMS in 72 participants with type 1 or 2 DM: 86.70% results in Zone A and 100% in Zones A + B of Consensus EGA^25^. Considering that percentages in Zones A + B represent accurate or acceptable glucose results^29^, we confirmed FGMS clinical usability in LC. Sensor clinical accuracy was not affected by factors such as gender, age, body mass index, Child-Pugh or edematoascitic decompensation, as the percentages of readings within Consensus Error Grid Zone A + B were persistently high (99.67–100%).

Even though user acceptability and satisfaction were not formally assessed, there were no unanticipated device associated side effects and the frequency of sensor insertion site related side effects (6.56%) was similar to the one reported by Scott *et al*. (7%)^21^.

The main limitation of this study was sample size, chosen mainly due to logistics and costs. Although data interpretation should have this in consideration, the design of the study with a control group minimize the loss of representativeness. Moreover, most studies approaching FGMS^22,25,30–34^ used sample sizes of 8 to 89 participants. Equally important, more patients with advanced stages of cirrhosis should be included.

In conclusion, this tool displayed a very satisfactory performance and usability in patients with LC and it sustained clinical accuracy in different stages of cirrhosis and in the presence of fluid overload, frequently observed in this population. This study provides promising preliminary evidence of sufficient performance and potential clinical usability to justify further randomized multicentric research.
